# Can school health education improve students’ physical exercise time? Empirical research based on CEPS (2014–2015) survey data

**DOI:** 10.3389/fpsyg.2022.1054275

**Published:** 2022-12-22

**Authors:** Huamei Zhong, Jingjing Zhou, Dan Xu, Tianbiao Liu

**Affiliations:** ^1^School of Physical Education and Sport Science, Fujian Normal University, Fuzhou, Fujian Province, China; ^2^School of Statistics, Beijing Normal University, Beijing, Beijing, China; ^3^Department of Physical Education, Beijing University of Posts and Telecommunications (BUPT), Beijing, Beijing, China; ^4^College of Physical Education and Sports Science, Beijing Normal University, Beijing, China

**Keywords:** physical exercise time, health perception, sports interest, school, health education

## Abstract

**Purpose:**

School health education is an effective strategy for cultivating adolescent physical exercise habits by transmitting healthy knowledge; it helps to form healthy behaviours and encourages students to participate in physical exercise. The purpose of this study was to explore the relationship between school health education and student participation in physical exercise.

**Methods:**

CEPS (China Education Penal Survey, 2014–2015) survey data were used to empirically analyse the impact of school health education on the time that students spend on physical exercise and the underlying mechanism of influence.

**Results:**

The results showed that receiving a school health education increased the time that students spent on physical exercise. 1) Compared with receiving health education in only primary school or secondary school, receiving health education in both primary and secondary school had a greater impact on students spending time on physical exercise. 2) Receiving a school health education improved the time that students spend on physical exercise by improving students’ health perception and their sports interests. 3) Receiving a school health education had a more obvious impact on the physical exercise time of male students, nonrural resident students, and students from multi-child households. These findings can provide a reference for the seasonable settings of health education curriculum in schools.

## 1. Preface

School health education is conducted through planned, organized, and systematic educational activities to encourage students to voluntarily change unhealthy behaviours and related factors that affect healthy behaviours, to eliminate or reduce risk factors that affect health, to prevent diseases, to promote health and to improve the quality of learning and life (Zou, 2008). School health education plays a positive role in promoting the physical and mental health of students. For this reason, many policies and curriculum standards have clearly promoted the development of school health education. In 2007, the State Council’s “Opinions on Strengthening Youth Sports and Strengthening the Physical Fitness of Young People” (Zhongfa [2007] No. 7) pointed out that it is necessary to carry out health education for adolescents and ensure adequate time for health education. In 2008, the Ministry of Education promulgated the “Guiding Guidelines for Primary and Secondary School Health Education” (Jiaotiyi [2008] No. 12), which clearly stipulated the specific goals and content of school health education. The “Healthy China 2030” Planning Outline also clearly stated that health education should be included in the national education system, and health education should be regarded as part of a high-quality education at all stages and important content in the curriculum (The Xinhua News Agency, 2016). At the same time, the “General High School Physical Education and Health Curriculum Standards (2017 Edition)” took health education as the core of the general high school physical education and health curriculum and emphasized the need to focus on physical education, integrate health education, and pay attention to moral education to cultivate students’ health awareness and behaviour and to promote the overall development of students (Ministry of Education of the People’s Republic of China, 2018).

Physical education and health curricula are important for the implementation of school health education. The common goal of both is to cultivate students’ healthy behaviours and promote the overall development of students’ physical and mental health. Therefore, the “Guiding Outline of Health Education for Primary and Secondary Schools” uses physical education and health courses as the main way to implement school health education. However, current school health education in China has problems in many aspects, which restrict the development of school health education courses, making it difficult for students to acquire health knowledge and develop healthy behavioural habits. In terms of health education and teaching, there is a lack of professional teachers, sufficient courses and teaching materials (You, 2014), and there is also a lack of implementation of courses and class hours (Fan et al., 2018). In the integration of health education and school physical education courses, the opening rate of health education part is low. In addition, school crowding has led to low levels of student health awareness and lack of awareness of the opportunity for autonomous participation. The health curriculum offered by physical education teachers has failed to cultivate students’ health awareness, behaviour, and lifestyle (Kong and Ping, 2019); surveys also show that health education classes are offered and held regularly at a percentage of 64.6% of all schools (Xu et al., 2014). The overall health education ability of PE teachers in Anhui Province is weak, and there are differences between urban and rural areas (Wang et al., 2018). The proportion of full-time health education teachers to schools in western Hunan is only 11.4% (Tian et al., 2019). In response to the problems of school health education, the “Healthy China 2030” Planning Outline clearly stipulates that health education should be included in the main content of preservice education and postservice training for physical education teachers. To give full play to the role of physical education and health curricula in implementing school health education and cultivating healthy behaviours in students, the Ministry of Education issued the “Opinions on Deepening the Integration of Sports and Education to Promote the Healthy Development of Adolescents” in August 2020, which aims to strengthen school physical education and improve youth fitness. The sports event system helps students enjoy having fun in physical exercise, strengthen their physique, improve their personality, exercise their will, and cultivate themselves as socialist builders and successors with the comprehensive development of moral, intellectual, physical, and artistic abilities (The Xinhua News Agency, 2020). To promote the construction of a healthy China, during the “14th Five-Year Plan” period, the state put school health education in a prominent position to improve the health literacy of young students. In August 2021, the Ministry of Education and five other departments jointly issued the “Opinions on Hygiene and Health Education” (Jiaotiyi [2021] No. 7). It proposed that the time reserved for school health education should be guaranteed by 2025, and the effect of health education teaching should be significantly improved. It also proposed to increase physical exercise time to ensure that students have 1 h of physical activity time inside and outside the school every day (Opinions on Comprehensively Strengthening and Improving School Health and Hygiene Education in the New Era, 2021).

In this context, with a view to promoting school health education courses, to fostering students’ healthy behaviours and to encouraging students to participate in physical exercise, we took use of the data from the China Education Tracking Survey (CEPS; 2014–2015) and conducted an empirical study regarding the impact of school health education on students’ physical exercise time and its underlying mechanism of influence.

## 2. Literature review and research hypotheses

School health education is an effective means of cultivating healthy behaviour by changing health cognition with knowledge to help form healthy living habits. Experimental intervention is the current mainstream method for studying the relationship between health education and physical exercise at home and abroad. The research mainly focuses on health education interventions for heterogeneous groups and explores the impact of health education on physical activities and healthy behaviours of different groups. Health education interventions in related studies target the general population, patients, disabled people, children and adolescents.

In the study of health education intervention for the general population and patients with chronic diseases, Young et al. (1996) showed that community health education had a positive impact on the percentage of men’s daily energy expenditure and time spent in strenuous activities. Liu et al. (2019) showed that after the intervention of health education for employees of a glove enterprise, adherence to the correct rate of “physical exercise 4 times a week or more” was significantly increased to 60.65%. Dorling et al. (2018) compared health education with “psychological inoculation” as follows: “The effect of intervention on self-reported physical activity, and the results show that psychological vaccination is more effective in increasing physical activity.” Health education intervention is also effective for increasing the physical activity of people with chronic diseases. The intervention results of Midhet et al. (2010) showed that the health education provided by Saudi Arabia’s primary health care centre can increase the physical activity level of patients with chronic diseases of all ages to a certain extent. A study by Lin and Wang (2017) showed that the excellent and good rates of physical exercise increased significantly after the implementation of a family synchronization health education intervention in elderly diabetic patients. Finally, related scholars have also conducted health education interventions for people with disabilities. Bodde et al. (2012) used health education interventions (video teaching, image memory tools, and interactive classroom activities) to demonstrate that the knowledge of physical activities of adults with mild to moderate intellectual disabilities would improve.

Scholars both at home and abroad have also used health education intervention experiments to study the impact of school health education on the physical activities of children and adolescents. The scholar Yannis started to intervene in children’s health education during the 1992–1993 school year, ended in the 1997–1998 school year, and followed up in the postintervention period in the 2001–2002 school year. The results after the intervention showed that the time of moderate to vigorous physical activities outside school in the intervention group was significantly increased (Manios et al., 1998). The follow-up results 4 years after the intervention showed that the intervention group’s leisure sports activities showed favourable changes (Manios and Kafatos, 2006), especially including activity by men. Postmoderate to high-intensity physical activity was significantly higher in the intervention period than in the four-year follow-up results, and moderate-to-high-intensity physical activity after the intervention and the four-year follow-up were 2.3 times and 2.1 times that of the control group among men, but there was no difference among women (Manios et al., 2006). This shows that health education intervention has a significant impact on children’s physical exercise and that it has a certain degree of sustainability. Subsequently, Manios and Kafatos (1999) implemented health and nutrition education interventions for children, Wang et al. (2000) implemented mental health education interventions for students, Harrison et al. (2006) conducted a 16-week health education intervention for primary school children, Christodoulos (2006) conducted health education interventions for Greek primary school students, Wang et al. (2012) conducted exercise health education interventions for primary school students in Guangzhou, and Efstathiou et al. (2016) conducted health education interventions for 10-year-old primary school students in Athens. Simbar et al. (2017) conducted skill-based health education interventions for female adolescents in Tehran, and Aittasalo et al. (2019) conducted health education interventions for Finnish eighth grade students. All the results indicated that health education intervention has a positive effect on the physical activity level of children and adolescents. In addition, research results have shown that health education classes can improve college students’ sports behaviours and increase leisure sports activities (Li and Wang, 2014; Sukys et al., 2019), and health education interventions can also improve the physical activities of high school students (Rezapour et al., 2016). Against the background of school health education reforms, high-intensity physical activity among primary school students also has a significant spillover effect on parents’ participation in light physical activity (Berniell et al., 2013).

In summary, health education experimental intervention research is the mainstream method for studying the relationship between health education and physical exercise both at home and abroad. Health education interventions are carried out for the general population, patients with chronic diseases, people with disabilities, children and adolescents, and college students, etc. The results of the interventions show that they increase their healthy behaviours. Education has an impact on physical activity or physical exercise and even has spillover effects on the physical activities of children’s parents. However, the relevant research seldom has seldom used social survey data to analyse the relationship between school health education and student physical exercise, nor has it explored the relevant mechanism of how school health education affects student physical exercise. Based on this, the use of microsocial survey data to study the impact of school health education on student physical exercise, to a certain extent, reduces the result deviation caused by uncontrollable factors in the experimental research. Second, this article uses measurement methods to estimate the impact of school health education on students’ physical exercise time. To solve the endogenous problems caused by missing variables and reverse causality, it also adopts the instrumental variable method, propensity score matching (PSM) and other methods. The study then makes estimates and provides references for follow-up related research. Finally, this article also discusses how receiving a school health education can increase students’ physical exercise time through a mechanism analysis.

School health education courses improve students’ health awareness through the transmission of health knowledge, which is conducive to cultivating their healthy behaviours and eliminating or reducing the impact of unhealthy factors on students’ physical and mental health. Physical exercise is an effective means to promote the physical and mental health of students in the context of health education. Relevant surveys show that 56% of students believe that the integration of physical education and health courses with health education is beneficial to their mastery of how to improve physical health. A total of 51.6% of students believe that health education courses were useful. This is conducive to their mastery of scientifically based physical exercise methods (Shen et al., 2011). At the same time, the results of the abovementioned school health education experimental interventions show that health education can improve the level of physical activity of students. Therefore, the first research hypothesis is proposed as follows: receiving a school health education improves the amount of time spent by students on physical exercise.

**Figure 1:**
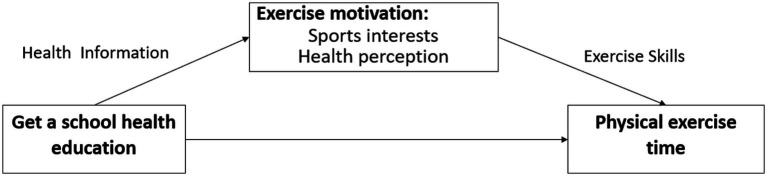
The “Information-Motivation-Behavioural Skills” model of school health education affects students’ physical exercise.

Physical exercises rely on sports skills to improve students’ healthy behaviours, and mastering sports skills is the basis for improving their physical health. The process of moving from receiving a school health education to the development of physical exercise behaviour can be attributed to the model of “information-motivation-behavioural skills.” This model was proposed by Fisher and Fisher (1992) and introduced self-efficacy theory on the basis of social cognitive theory, and divided the factors that affect individual behaviour into information, motivation and behavioural skills. The “information-motivation-behaviour skills” model believes that the decisive factors affecting behaviour should be sought from the three levels of information, motivation, and behaviour skills, interventions should be implemented, and the effect of motivation and behaviour skills on behavioural change should be emphasized (Fisher et al., 1996). Receiving a school health education makes students have exercise motivation by transmitting health information and develops physical exercise behaviours by mastering sports skills. The self-health perception generated by students who receive health knowledge information is an important source of exercise motivation. At the same time, related studies argue that sports learning interest is an important variable affecting motivation and student willingness to participate in learning sports and physical exercise (Lin, 2019). Therefore, in the school education system, the motivation of students to exercise mainly comes from receiving a school health education, transmitting health information to improve their individual health perception and sports interests and then using the mastery of motor skills as a health-promoting behavioural skill to drive students to spend more time participating in physical exercise to improve physical fitness (see Figure 1). Based on this, the second research hypothesis is put forward as follows: students who receive a school health education increase their physical exercise time by improving their individual sense of health perception and sports interests.

## 3. Research methods, data sources and variable descriptions

### 3.1. Research methods

#### 3.1.1. Benchmark regression model

To investigate the impact of school health education on students’ physical exercise time, the benchmark regression Equation (1) was established. In the equation, *lnExercise_i_* is the average daily physical exercise time, *Health_edu_i_* is whether school health education was received, and *Control_i_* is a control variable such as individual, school, family, etc.


(1) $${\text{lnExercise}}_{i}=\alpha_{0}+\alpha_{1}\text{Health}\_{\text{edu}}_{i}+\gamma {\text{XControl}}_{i}+\varepsilon$$

#### 3.1.2. Robustness test

The data in this study are cross-sectional. Propensity score matching, quantile regression and merge regression equations are used to test the robustness according to the characteristics of the data. ① Propensity score matching (PSM) was proposed by Rosenbaum and Rubin (1983) and is a measure of the matching distance between individuals. The steps include incorporating the relevant variables that affect physical exercise time and school health education into the model, first using logit regression to estimate the conditional probability (propensity score) of whether students receive a school health education, and then testing the balance of the matching results in the treatment group and the control group. Finally, nearest neighbour matching, radius matching, and nuclear matching are used to calculate the average treatment effect (ATT) of receiving a school health education. ② To eliminate the influence of extreme values on the estimation results, according to the quantile regression proposed by Koenker and Hallock (2001), the weighted average of the absolute value of the residual is used as the minimization objective function. Regression was performed using the 25, 50, and 75% quantiles to judge the robustness of the research results. ③ The value of daily physical exercise time of students can only be nonnegative numbers, and the daily physical exercise time of some students is 0, showing the characteristics of truncated data with 0 as the left merge. Therefore, the daily physical exercise time is used as the dependent variable to use Tobit to retest the robustness of the research results.

#### 3.1.3. Mechanism inspection

To test whether physical interest and health perception are the mediating mechanism of school health education affecting students’ physical exercise time, the mediation effect test method of Wen et al. (2004) was established to create Equation (3) and Equation (4), and bootstrapping was adopted. The intermediary effect test method carries out direct effect and indirect effect tests.


(2) $${\text{Mediation}}_{i}=\beta_{0}+\beta_{1}\text{Health}\_{\text{edu}}_{i}+\gamma {\text{XControl}}_{i}+\varepsilon$$

(3) $$\begin{array}{l} {\text{lnExercise}}_{i}=\delta_{0}+\delta_{1}\text{Health}\_{\text{edu}}_{i}+\delta_{2}{\text{Mediation}}_{i}\\ \;\;\;\;\;\;\;\;\;\;\;\;\;\;\;\;\;\;\;\;\;\;\;\;\;\;\;\;\;\;\;+\gamma {\text{XControl}}_{i}+\varepsilon \end{array}$$

In the equation, Mediationi is an intermediate variable, including sports interest and health perception. The regression coefficient relationship in Equation (1), Equation (3), and Equation (4) is *α_1_ = δ_1_ + β_1_ × δ_2_*, where *α_1_* is the total effect, *δ_1_* is the direct effect, and *β_1_ × δ_2_* is the indirect effect.

### 3.2. Data sources and variable description

This research uses China Education Panel Survey (CEPS) 2014–2015 microsurvey data. The CEPS is designed and implemented by the Survey and Data Center of Renmin University of China. It is a nationally representative education follow-up survey that objectively reflects the relationship between the demographic structure of our country’s family, school and other social units and personal education output. The 2014–2015 year was the year of the first follow-up survey, with a total of 10,750 student samples. According to the research design, the student survey data were matched with school and family data. After matching, the data were cleaned, and the missing values were eliminated. Finally, 6,983 valid samples were obtained.

According to the research design, the main variables of this paper include dependent variables, independent variables, control variables and intermediate variables (see Table 1).

**Table 1:** Variable names, definitions, and descriptive statistics.

Variable name	Variable definition	Mean value/ Proportion(%)	Standard deviation
**Dependent variable**			
Physical exercise time	Average daily physical exercise time + 1, taking the logarithm	2.733	0.911
**Independent variables**			
School Health Education	Not accepted = 0, accepted = 1	77.40%	
School health education section	Not accepted = 1, elementary school only = 2, junior high school only = 3, elementary school + junior high school =4	2.905	1.241
**Control variables**			
Gender	Female =0, Male =1	51.25%	
Household registration	Agricultural household =0, nonagricultural household =1	46.26%	
Only child	Non-only child =0, only child =1	43.86%	
Cognitive ability	Standardized score on cognitive ability test	0.321	0.792
Media use	The average media use time per day +1, taking the logarithm	3.402	1.877
Nature of the school	Private school =0, public school =1	92.20%	
Training funds per student	Financial allocations per student +1, taking the logarithm	6.623	1.293
Sports field	No =0, yes =1	98.02%	
Gymnasium	No =0, yes =1	19.45%	
Swimming pool	No =0, yes =1	2.49%	
Family financial conditions	Very difficult =1, relatively difficult =2, medium =3, relatively rich =4, very rich =5	2.802	0.608
Father’s education level	Years of education	10.39	3.137
Mother’s education level	Years of education	9.770	3.524
**Mediating variables**			
Health perception	Very bad =1, not so good =2, general =3, better =4, very good =5	3.868	0.927
Sports interest	No =0, yes =1	35.89%	

#### 3.2.1. Dependent variable

Improving exercise awareness, forming exercise habits and actively participating in physical exercise are the concrete manifestations of students’ health behaviour literacy. The length of student physical exercise time can objectively reflect the impact of school health education on healthy student behaviour. Using the method of Hu and Yu (2019) the student physical exercise time is defined as the average daily physical exercise time.^1^ To keep the sample with 0 physical exercise times and make the variables conform to the normal distribution, the physical exercise time is the average daily physical exercise time + 1, and the logarithm is taken.

#### 3.2.2. Independent variables

The core explanatory variable in this article is school health education because the students in the questionnaire received school health education classes, including whether courses were taken through elementary school and junior high school^2^ or just in elementary school or junior high school. Receiving health education was indicated as receiving a school health education, and the rest are marked as not receiving a school health education. Namely, school health education: unaccepted = 0, accepted = 1, which are dummy variables; at the same time, to analyse the impact of receiving health education at different stages on students’ physical exercise, a sequenced variable school health education section is constructed.

**Figure 2:**
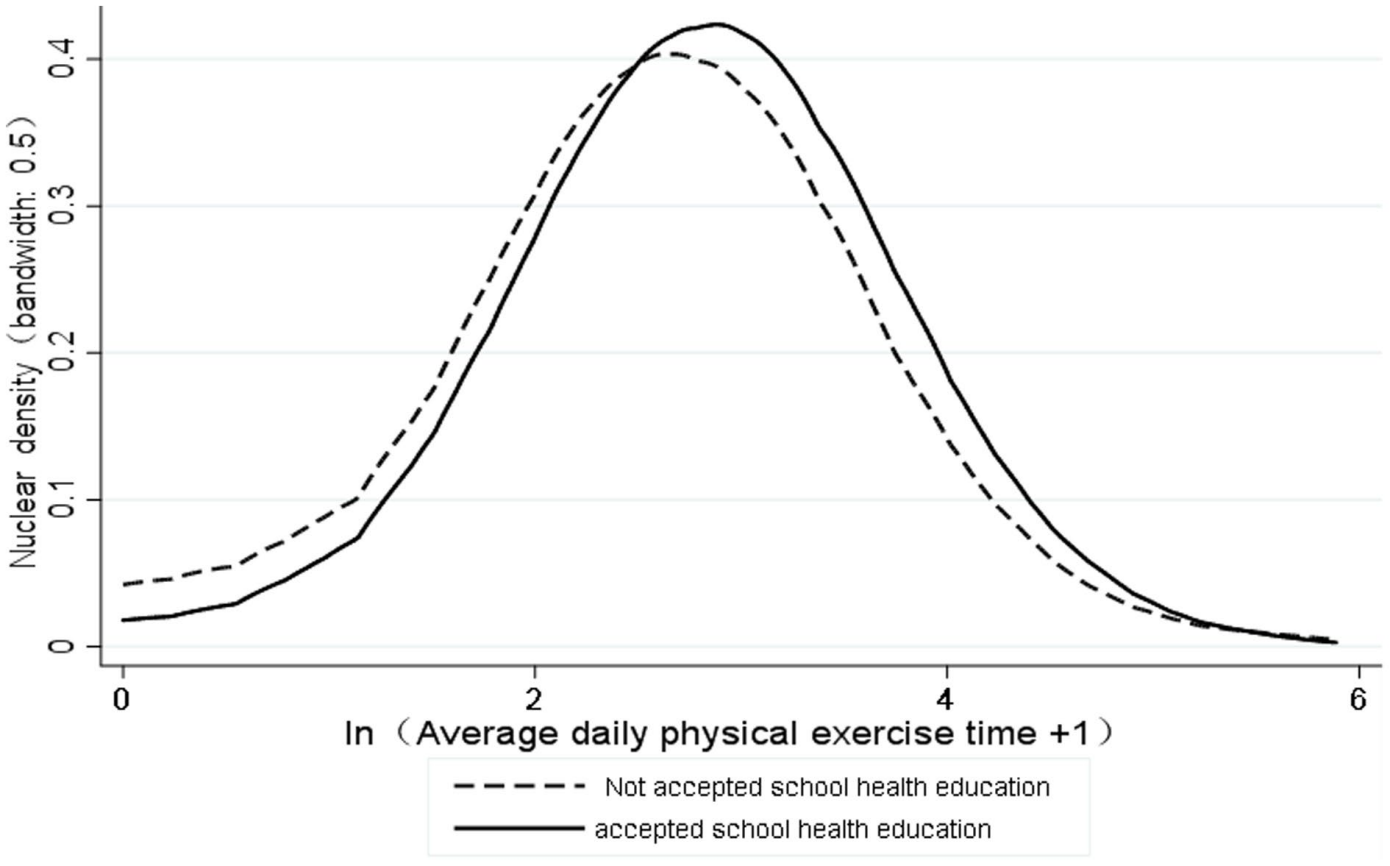
Distribution of kernel density estimates for average daily physical exercise time.

The Figure 2 reports the kernel density estimation of the average daily physical exercise time of students who received a school health education and those who did not. As for distribution, the nuclear density peak of students who receive a school health education was significantly higher than that of students who did not receive a school health education, which indicates that the average daily physical exercise time of students who receive a school health education was significantly higher than that of students who did not receive a school health education.

#### 3.2.3. Control variables

First, this article controls for the individual demographic characteristics of students. At the same time, research also shows that internet addiction (IA) has an impact on students’ physical health (Liu, 2016). The lower the cognitive ability of adolescents is, the higher their risk of health-risk behaviours is (Wang et al., 2019). Therefore, student gender, household registration, only child status, media use, and cognitive ability are used as individual control variables. Second, school factors that affect students’ physical exercise include the nature of the school and school sports facilities, and family factors include parents’ education level and family economic status (Hu and Yu, 2019). At the same time, the school’s financial status can reflect the quality of student training and provide conditions for students to participate in physical exercise. Therefore, the school-level control variables include school nature, per-student training funds, sports fields, gymnasiums, and swimming pools as control variables. At the family-level, family economic conditions, father’s education level and mother’s education level were used as control variables.

#### 3.2.4. Intermediary variables

According to the above research hypothesis, students’ health perception and sports interests may be the source of motivation for students to receive a school health education for physical exercise. Therefore, the students’ self-perceived health status is selected to measure their health perception; sports interest is measured by a binary variable, that is, whether there is interest in sports.

## 4. Empirical analysis of the impact of school health education on student physical exercise

### 4.1. Benchmark regression estimation

Columns (1)–(3) of Table 2 present the benchmark regression results of the impact of school health education and school health education on physical exercise time. The model test results show that all models are significant and that the independent variables are multicollinear. The test results show that the VIF value of each variable is less than 10, which indicates that there is no multicollinearity among the independent variables.

**Table 2:** The impact of school health education on students’ physical exercise.

Independent variable	OLS		
	Physical exercise time		
	(1)	(2)	(3)
School health education	0.228*** (0.028)	0.181*** (0.027)	
School health education section			
Elementary school only			0.118*** (0.036)
Junior high school only			0.110*** (0.038)
Elementary school + junior high school			0.220*** (0.029)
Gender		0.184*** (0.021)	0.185*** (0.021)
Household registration		0.056** (0.026)	0.056** (0.026)
Only child		0.083*** (0.025)	0.083*** (0.025)
Cognitive ability		0.103*** (0.016)	0.103*** (0.016)
Media use		0.004 (0.006)	0.004 (0.006)
School nature		−0.112** (0.045)	−0.125*** (0.046)
Sports field		0.060*** (0.011)	0.058*** (0.011)
Gymnasium		−0.128 (0.098)	−0.132 (0.099)
Swimming pool		0.087*** (0.031)	0.090*** (0.031)
Average training expenditure per student		0.328*** (0.064)	0.304*** (0.064)
Family financial conditions		0.040** (0.020)	0.039* (0.020)
Father’s education level		0.009* (0.005)	0.009* (0.005)
Mother’s education level		0.012*** (0.004)	0.012*** (0.004)
constant	2.557*** (0.025)	1.865*** (0.127)	1.893*** (0.128)
Prob>F	0.000	0.000	0.000
R2	0.011	0.072	0.074
Observations	6983	6983	6983

***, **, and * indicate significance in 1, 5, and 10% confidence intervals, respectively, with a robust standard error in parentheses.

Columns (1) and (2) of Table 2 are the regression results of not adding and adding control variables, respectively. The regression coefficients of school health education on physical exercise time are 0.228 and 0.181, and they are both significant in the 1% confidence interval, which indicates acceptance. The average daily physical exercise time of school health education students was 18.1% higher than that of students who did not receive health education. The first research hypothesis is established; that is, receiving a school health education increases students’ physical exercise time. To analyse the impact of receiving a school health education in different stages on students’ physical exercise time, Column (3) of Table 2 introduces school health education stage variables to analyse the impact of receiving a school health education in different school stages on students’ physical exercise time. The results showed that compared with students who did not receive a school health education, the average daily physical exercise time of students who received a school health education only in elementary school, those received a school health education only in middle school, and those who received a school health education in both primary and middle school increased by 11.8.%, 11.0, 22.0%, respectively, and the results are all significant within the 1% confidence interval. It can be seen from the size of the regression coefficient that compared with students who receive a school health education only in elementary school and junior high school, students who receive a school health education in both elementary and junior high school have a more obvious effect on improving their physical exercise time.

### 4.2. Robustness test

According to the above robustness test steps, we first use propensity score matching (PSM) for the robustness test. The first step is to use school health education as the explanatory variable and select gender, household registration, only child status, cognitive ability, media use, school nature, training funds per student, and the presence of a sports field, gymnasium, or swimming pool as explanatory variables to perform logit regression and estimate the propensity score; in the second step, neighbour matching, radius matching, and kernel matching are performed. The balance test results show that the variance of variables after matching is less than 10%. Table 3 presents the matching results. The average treatment effect ATT values of the three matching methods are 0.171, 0.183, and 0.187 and are significant at the 1% confidence interval. The average of the three matching methods is 0.180; that is, the daily average of how much time students who are receiving a school health education spend on physical exercise is 18.0% higher than that of students who have not received a school health education, which is not much different from the 18.1% estimated by OLS.

**Table 3:** Robustness test: propensity score matching (PSM).

Match method	Accepted school health education	Did not accept school health education	ATT	Standard error	T value
Neighbour matching^a^	2.783	2.612	0.171***	0.033	5.150
Radius matching^b^	2.782	2.599	0.183***	0.031	5.840
Core matching	2.783	2.595	0.187***	0.031	6.130

a: one-to-four nearest neighbour matching with replacement is used; b: the radius is 0.01; the three matching methods successfully match 6984, 6962 and 6964 samples, respectively; *** means significant in the 1% confidence interval, namely, the T value is greater than 2.58.

This article again uses quantile regression to test the robustness (see Table 4), and the results show that at the 25, 50, and 75% quantiles, school health education has a significant positive impact on students’ physical exercise time. The robustness test was carried out using the Tobit method. The regression results showed that the average daily physical exercise time of students who receive a school health education increased by 18.9%. The robustness results once again show that receiving a school health education improves students’ physical exercise time.

**Table 4:** Robustness test: quantile regression, merge regression.

Independent variable	Physical exercise time (QR)			Physical exercise time (Tobit)
	q25	q50	q75	
School health education	0.187*** (0.045)	0.106*** (0.027)	0.096*** (0.034)	0.189*** (0.026)
Control variable	Control	Control	Control	Control
Constant	1.301*** (0.166)	2.037*** (0.104)	2.588*** (0.172)	1.836*** (0.107)
Pseudo *R*^2^	0.032	0.057	0.063	0.027
Observations	6983			

QR regression brackets are bootstrap standard errors, and Tobit regression brackets are standard errors. ***, **, and * indicate significance in the 1, 5, and 10% confidence intervals, respectively, and the robust standard errors are in parentheses.

### 4.3. Test of the intermediary mechanism of school health education affecting students’ physical exercise time

According to the intermediate effect test equation established above, the nonparametric percentile bootstrap test method of deviation correction adopted by Yang et al. (2020) was used for the intermediate effect test, with sampling repeated 1,000 times and the direct confidence interval calculated at 95%. Direct effect and indirect effect values were assigned to verify whether receiving a school health education affects physical exercise time by improving students’ health perception and sports interests. The results show (see Table 5) that the indirect effect (*β_1_ × δ_2_*) of increasing students’ physical exercise time and improving students’ health perception by receiving a school health education is 0.012, the proportion of the indirect effect to the total effect (δ*_1 + 1_β × δ_2_*) is 6.63%, and the 95% confidence interval is [0.006,0.019] and excludes 0. The indirect effect (*β_1_ × δ_2_*) of increasing students’ physical exercise time and improving students’ health perception by receiving a school health education is 0.019, and the indirect effect accounts for the total effect (the proportion of δ*_1 + 1_β × δ_2_*) at 10.50%, with a 95% confidence interval at [0.009,0.029] that excludes 0. At the same time, health perception and sports interests are part of the mediating role. In summary, receiving a school health education increases students’ physical exercise time by improving students’ health perception and sports interests. The second research hypothesis has been verified.

**Table 5:** Test results of mediation effect based on Bootstrap method.

Intermediary variables		Coefficients	Bootstrap Standard error	95% confidence interval		Intermediary degree
				Lower limit	Upper limit	
Health perception	Indirect effect	0.012	0.003	0.006	0.019	6.63%
	Direct effect	0.169	0.026	0.117	0.221	
Sports interests	Indirect effect	0.019	0.005	0.009	0.029	10.50%
	Direct effect	0.162	0.026	0.112	0.213	

The number of times of Bootstrap resampling is 1000.

### 4.4. Analysis of the heterogeneity of school health education affecting students’ physical exercise time

Table 6 presents the heterogeneity of school health education affecting students’ physical exercise time. The subsample estimation results of gender, household registration, and only child status show that receiving a school health education has improved the physical exercise time of students of different genders, students with different household registrations, and students from both only-child and multi-child households, and all are significant within the confidence interval of 5% or more. From the perspective of regression coefficients, receiving a school health education increased the physical exercise time of female students, agricultural household registration students, and only-child students by 8.1, 16.3, and 13.4%, respectively. Overall, per each regression coefficient, physical exercise time increased by 26.7, 20.6, and 21.7%, which indicates that a school health education has a more obvious effect on increasing the physical exercise time of male students, nonagricultural students and students who are not only-children.

**Table 6:** Heterogeneity of school health education affecting students’ physical exercise time.

Self-variable	Physical exercise time (OLS)					
	Gender		Household registration		Whether the student is an only-child	
	Female	Male	Agricultural households	Non-Agricultural households	Non-only child	Only child
School health education	0.081** (0.033)	0.267*** (0.042)	0.163*** (0.036)	0.206*** (0.042)	0.217*** (0.035)	0.134*** (0.043)
Control variable	Control	Control	Control	Control	Control	Control
constant	1.939*** (0.162)	2.072*** (0.187)	2.073*** (0.162)	1.471*** (0.216)	1.788*** (0.160)	1.973*** (0.200)
Prob>F	0.000	0.000	0.000	0.000	0.000	0.000
*R* ^2^	0.086	0.058	0.046	0.077	0.056	0.069
Observations	3404	2579	3753	3230	3920	3063

***, **, and * indicate significance in the 1, 5, and 10% confidence intervals, respectively, and the robust standard errors are in parentheses.

## 5. Discussion

The empirical results show that receiving a school health education increases students’ physical exercise time, which indicates that deepening school health education can be an effective means to improve the physical health of adolescents. At the same time, the sooner the school health education is carried out with cohesion between semesters, the more effective the school health education will be in improving the healthy behaviour of students. Adolescent obesity and myopia caused by insufficient physical activity have become key factors that affect the healthy growth of adolescents. The results of the 8th National Student Fitness and Health Survey show that in 2019, the national 6-to 22-year-old student’s physical health compliance rate was 23.8%. The rate of junior middle school students who achieve 1 h of physical exercise per day is only 42.7%, and the rate of good physical fitness is only 29.2% (Department of Physical Health and Arts Education, 2021). The results of monitoring the national compulsory education quality in 2018 showed that the detection rate of poor eyesight in eighth grade students reached 68.8%, and the detection rate of poor eyesight in urban schools was higher than that in rural schools (2018 Reports of National Quality Monitoring of compulsory education in mathematics, 2018). To curb the decline in the physical health of young people and promote their healthy growth has become a key task in the construction of a healthy China. Increasing physical exercise time is the key to solving a series of health problems for adolescents. The role of school health education in promoting students to participate in physical exercise time is prominent. The reason can be attributed to the fact that the establishment of school health education courses has effectively improved the health literacy of students. School health education teaches knowledge about healthy behaviours and lifestyles, disease prevention, and mental health to students at different stages to improve their health literacy. Participating in physical exercise is an important manifestation of healthy behaviour and lifestyle. Students who receive a school health education improve their health literacy and internalize it into physical exercise behaviour, thereby increasing their physical exercise time expenditure.

Meanwhile, students’ self-health perception and sports interests are the key motivations for students’ physical exercise behaviours. However, the overall health literacy of students in our country is low, and the conversion rate of sports interests to exercise behaviours is low. A sample survey of student health literacy in Beijing in 2020 shows that the overall performance of Beijing’s primary and middle school students in daily hygiene and health behaviours and habits needs to be improved. The main reason for the low health literacy of students is the weakness of school health education. Health education lacks a strong management team, the curriculum implementation mode is relatively singular, and teacher resource support is lacking (Xu, 2020). The results of the national compulsory education quality test in 2018 showed that the proportions of eighth grade students in the country who like physical education, physical education teachers and extracurricular sports activities were 89.6, 88.0, and 79.6%, respectively. A total of 1.5% of students refused to participate in sports activities, but only 18.6% of eighth graders had physical exercise habits (2018 Reports of National Quality Monitoring of compulsory education in mathematics, 2018), which indicate that the rate of conversion of interest in sports to physical exercise behaviours among adolescent students in China is relatively low. Therefore, there is an urgent need to strengthen school health education to enhance students’ self-perception of health and sports interests. Internalizing health perception and sports interests into adolescents’ own physical exercise habits is a powerful measure to improve the physical health of young people.

In terms of the influence of school health education on physical exercise behavior of different groups of students, the participation rate of male students in junior high school is higher than that of female students. Female students have earlier physical development and higher rates of mental health problems caused by changes in physical characteristics, making their participation rate in physical exercise lower; most students with agricultural household registration are studying in rural middle schools, such schools may not have enough P. E teachers and sports facilities which restrict their participation in physical exercises; the only-child receives too much attention from their parents and devotes more time to the study of other subjects, so that less physical exercise time is allocated. Therefore, in the context of promoting education equity, the state promulgated and implemented the “Opinions on Further Reducing the Burden of Students’ Work and Extra-School Training in Compulsory Education” and the “Opinions on Comprehensively Strengthening and Improving School Hygiene and Health Education in the New Era” (Education Art [2021] No. 7), which aims to reduce the academic burden of students and create favourable conditions for promoting the physical and mental health of young people. Therefore, school health education urgently needs to focus on relatively disadvantaged groups, such as female students, agricultural household registration students, and only-child students. The health education curriculum should reflect individual differences. At the same time, it is necessary to improve the sports facilities in rural middle schools to provide fair education for different groups of students. Opportunities should be equitable, so that students have the opportunity to receive health education and cultivate physical exercise habits.

## 6. Conclusion

School health education is conducive to the physical and mental health of students, and school physical education is an important carrier of school health education. Physical education and health courses have become the main means of school health education by promoting the integration of school health education and school physical education. This article explores the impact of school health education on students’ physical exercise time and its mechanism by using data from the China Education Tracking Survey (CEPS; 2014–2015) for empirical analysis. The empirical results obtained the following conclusions: receiving a school health education increased students’ physical exercise time, using the instrumental variable method and propensity score matching (PSM), and quantile regression and merging regression for robustness testing all obtain the same conclusion. The estimation results show that compared with students who only receive health education in elementary school or middle school, students who receive a school health education in both elementary and middle school have a greater impact on their physical exercise time. The test results of the mediation effect show that receiving a school health education improves students’ physical exercise time by improving students’ health perception and sports interests. The heterogeneity test results show that receiving a school health education has a greater impact on male students, nonagricultural household registration students, and non-only-child students’ physical exercise time. In response to the above research conclusions, the following suggestions are made.

First, it is necessary to promote the integration of school health education and school sports from a policy perspective. Health education and school physical education have a high degree of consistency in the pursuit of the intrinsic value of “promoting health”(Kong and Ping, 2019), and school health education can encourage students to participate in physical exercise by improving their health perception and sports interests. Improving the physical health of young people is the key goal and an important task of current school sports development. Under the influence of the pressure of entering school and social environmental factors, young people frequently have low health literacy and a lack of interest in sports. Therefore, relevant policies should actively promote the integration of school health education and school physical education. School health education courses should cultivate healthy student behaviours from the perspective of popularizing health knowledge to promote their active participation in physical exercise, meaning that school health education courses should be included with physical education. Health courses are an effective supplement to healthy behaviours. At the same time, in the integration of school health education and school sports, it is necessary to strengthen the health education strategies for female students, rural household registration students, and only child students to guide these groups to actively participate in physical exercises to promote physical and mental health.

The second is to increase the time for school health education courses and ensure both quantity and quality of teachers for school health education courses. At present, the physical education and health curriculum is the basis of the implementation of health education, but the physical education and health curriculum has limitations in terms of time and teachers (Liu, 2019). Therefore, in terms of teaching time, on the one hand, it is necessary to ensure that the health education hours in the physical and health courses are properly allocated; on the other hand, it is necessary to use school-based courses, large breaks, and extracurricular activities to carry out school health education. In terms of teachers, colleges and universities, the training of physical education teachers should include the curriculum of health education and make a good reserve of health education teachers. Physical education teachers in primary and secondary schools should strengthen their training and seek re-education in the health education knowledge system to better meet the needs of health education in primary and secondary schools.

The third is to strengthen the systematization of school health education knowledge and better link the health education content of each school segment. Studies have shown that there is cohesion between the school stages in which students receive health education and an improved effect of school health education in promoting students’ physical exercise. Therefore, it is advisable to carry out school health education sooner rather than later, and the school health education curriculum for each school stage should reflect individual differences and be cohesive. Regarding the setting of health education courses, schools of all levels and types should set up health education courses as early as possible and in a timely manner. On the other hand, they need to promote the connection of health education courses between different semesters so that students can receive school health as early as possible and continuously. Education, through school health education, cultivates healthy student behaviours to give full play to the educating function of sports.

The limitations of this study include the failure to control more relevant factors such as family, community and society that affect students’ physical exercise time, as well as failure to find effective instrumental variables and conduct experimental studies to estimate the causal relationship between “received school health education” and “time of physical exercise.” In future research, it is hoped to further clarify the causal relationship between “school health education” and “physical exercise time” through experimental intervention studies. Moreover, it is also necessary to analyze the relationship between school health education and other healthy lifestyle formations, such as diet, smoking, and drinking.

## Data availability statement

Publicly available datasets were analyzed in this study. This data can be accessed through http://ceps.ruc.edu.cn/.

## Ethics statement

Ethical review and approval were not required for the study on human participants in accordance with the local legislation and institutional requirements. Written informed consent from the [patients/ participants OR patients/participants legal guardian/next of kin] was not required to participate in this study in accordance with the national legislation and the institutional requirements.

## Author contributions

HZ conceptualized the study, wrote the original draft preparation, and contributed to the methodology and data collection. TL reviewed and edited the manuscript and helped to improve this work. JZ helped to edit the manuscript and reviewed this work. DX reviewed this work. All authors contributed to the article and approved the submitted version.

## Funding

This study was supported by the National Social Science Fund of China (18CTY011).

## Conflict of interest

The authors declare that the research was conducted in the absence of any commercial or financial relationships that could be construed as a potential conflict of interest.

## Publisher’s note

All claims expressed in this article are solely those of the authors and do not necessarily represent those of their affiliated organizations, or those of the publisher, the editors and the reviewers. Any product that may be evaluated in this article, or claim that may be made by its manufacturer, is not guaranteed or endorsed by the publisher.

## Footnotes

^1^Questionnaire Item C13: Time spent on physical exercise: usually several days per week and how many minutes per day. Average daily physical exercise time = (how many minutes of physical exercise per week)/7.

^2^The questionnaire item is C20: Did you take a health education class when you were in elementary school? C21: Have you ever attended a health education class after entering junior high school?
